# Relationship between iliopsoas muscle surface pressure and implant alignment after total hip arthroplasty: a cadaveric study

**DOI:** 10.1038/s41598-023-30734-5

**Published:** 2023-03-01

**Authors:** Yasuaki Tamaki, Tomohiro Goto, Joji Iwase, Keizo Wada, Yasuyuki Omichi, Daisuke Hamada, Yoshihiro Tsuruo, Koichi Sairyo

**Affiliations:** 1 Department of Orthopedics, Institute of Biomedical Sciences, Tokushima University Graduate School, 3-18-15 Kuramoto, Tokushima, 770-8503 Japan; 2 Department of Anatomy and Cell Biology, Institute of Biomedical Sciences, Tokushima University Graduate School, Tokushima, Japan

**Keywords:** Diseases, Medical research, Signs and symptoms

## Abstract

Iliopsoas impingement after total hip arthroplasty is caused by the implant irritating the iliopsoas muscle, but changes in the iliopsoas muscle have not been quantitatively evaluated. This study assessed changes in the surface pressure of the iliopsoas muscle when the implant alignment was varied. Total hip arthroplasty was performed in 10 fresh-frozen cadaveric hips. We evaluated the maximum and mean surface pressure of the iliopsoas muscle with the hip in 20° and 10° extension, the neutral position, and 10° flexion when the anterior cup protrusion length (ACPL), stem version, and stem offset were varied. When the ACPL was changed to 0, 3, and 6 mm in 20° extension, the maximum surface pressure was significantly increased for ACPL of 6 mm compared with 0 mm. Decreased stem anteversion resulted in a significant reduction in both the maximum and mean surface pressure compared with native anteversion from 20° extension to the neutral position. Increased stem offset resulted in significant increases in the maximum and mean surface pressure of the iliopsoas muscle compared with decreased stem offset in 20° extension. Not only large ACPL but also changes in stem version and offset affected the maximum surface pressure of the iliopsoas muscle.

## Introduction

Iliopsoas impingement (IPI) is a well-known cause of groin pain after total hip arthroplasty (THA).^1–7^ The reported incidence of symptomatic IPI after THA ranges from 0.45 to 4.6%^4–9^. Symptomatic IPI typically occurs during active flexion or passive extension of the hip and causes anterior groin pain during activities of daily living such as ascending stairs or riding in a vehicle^7,10,11^. The initial management of IPI is generally conservative treatment, including physical therapy, non-steroidal anti-inflammatory drugs, and iliopsoas tendon sheath corticosteroid injections. However, when conservative treatment is ineffective, surgical treatment is necessary, such as iliopsoas tenotomy or revision surgery of the acetabular cup^1,2,12^. IPI is a major concern after THA because of its negative impact on postoperative ADL and risk of reoperation.

Several clinical studies have described the etiology and pathogenesis of IPI after THA. It is well known that mechanical irritation of the iliopsoas muscle by the protruding part of the anterior cup is the main cause of symptomatic IPI^1,3,4,10,13^. In addition, decreased cup anteversion, increased stem offset, large stem anteversion, and increased leg length have been reported as potential risk factors of symptomatic IPI^3,11,14–17^. We speculated that changes in implant alignment may cause changes in the surface pressure of the iliopsoas muscle, which may lead to pain. However, to our knowledge, changes in the surface pressure of the iliopsoas muscle at the site of impingement after THA have not yet been quantitatively evaluated.

The purpose of this cadaveric study was to directly measure changes in the surface pressure of the iliopsoas muscle using a seat-type pressure sensor when the anterior cup protrusion length (ACPL), stem anteversion, and stem offset were varied during THA.

## Methods

### Subjects

This study was approved by the Institutional Review Board of Tokushima University Graduate School (approval no. 2068) and performed in accordance with the principles of the Declaration of Helsinki. Written informed consent is routinely obtained from all cadaver donors and their families when they donate their bodies to the institution for research purposes.

Ten hips of 8 fresh-frozen whole-body cadavers were used in this study (male: 6 hips; female: 4 hips). Evaluation was performed bilaterally in 2 cadavers and unilaterally in the remaining 6 cadavers. Mean age at the time of death was 79.2 (range, 65–96) years. All cadaveric specimens were macroscopically intact without gross deformity or obvious joint contracture. In all the specimens, two senior hip surgeons evaluated the osseous morphology (joint space narrowing, osteophytes, etc.) according to the Tonnis classification on computed tomography (CT) coronal sections^18^. We excluded hip osteoarthritis of grade 2 or 3, past history of hip surgery and excessive scoliosis or kyphosis evaluated on CT sagittal sections. All cadavers were stored at -20 °C until examination. Before use, each specimen was thawed for 48 h at 21 °C (room temperature).

### Surgical procedure

**Figure 1:**
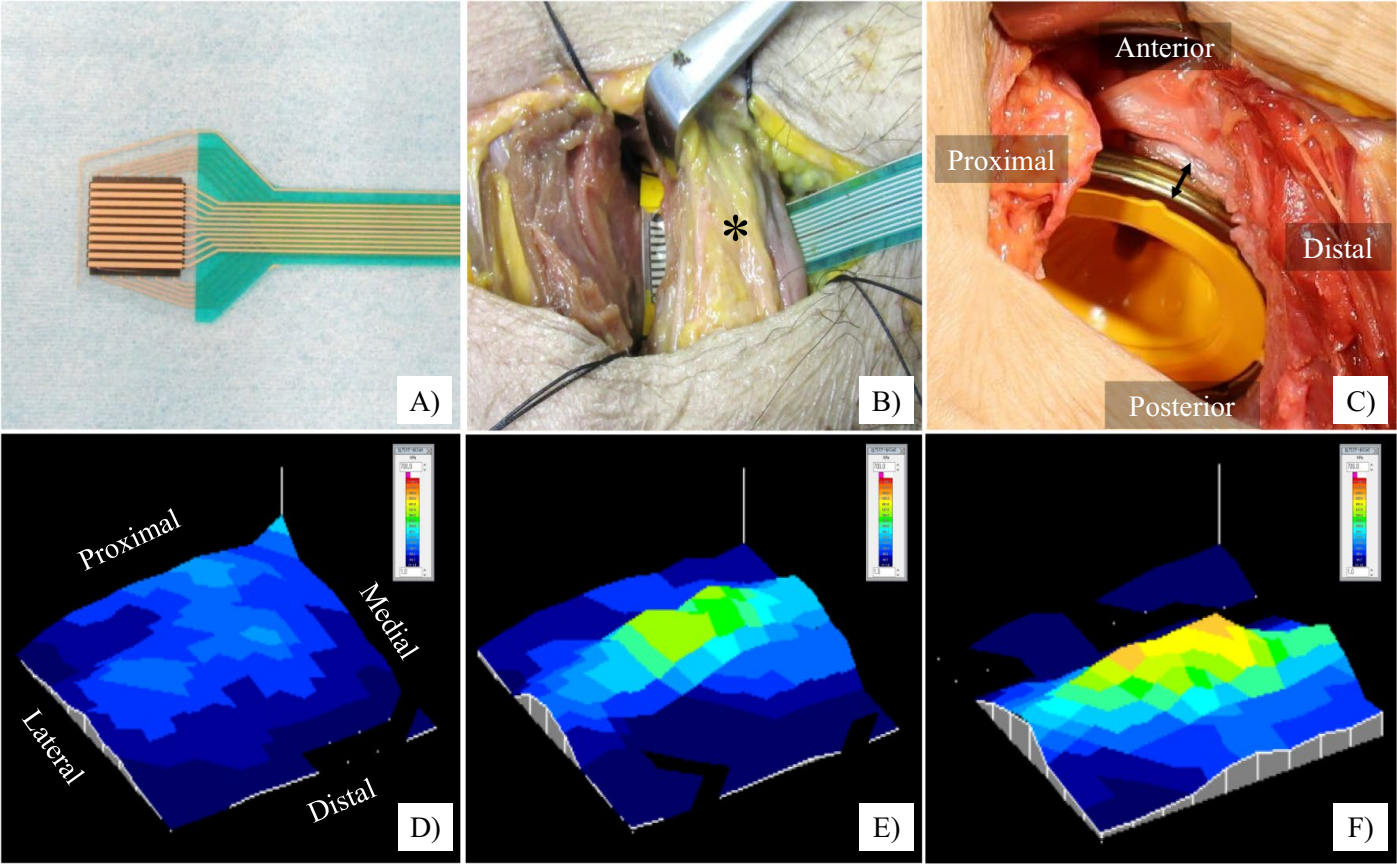
Photographs of the seat-type pressure sensor for real-time monitoring of the surface pressure of the iliopsoas muscle (I-SCAN, Nitta, Osaka, Japan). (**A**) The sensor is 0.1 mm thick with dimensions of 10 × 10 mm. (**B**) The pressure sensor was placed between the iliopsoas muscle and anterior cup edge via an ilioinguinal approach. (**C**) Photograph showing the condition of the anterior cup protrusion length. (**D**–**F**) A typical case of the maximum surface pressure of the iliopsoas muscle with the hip in 20° of extension. Anterior cup protrusion length: (**D**) 0 mm, (**E**) 3 mm, (**F**) 6 mm.

We performed THA in the supine position with a G7 OsseoTi cup (Zimmer Biomet, Warsaw, IN) as the trial cup and a femoral rasp for the Wagner cone hip stem (Zimmer Biomet) using an anterolateral approach. We made a skin incision at the anterior border of the gluteus medius muscle and accessed the hip joint through the interval between the tensor fascia lata and gluteus medius. We used a CT-based navigation system (Stryker, Freiburg, Germany) for preoperative planning, implantation, and monitoring of the hip position during examination. We resected the femoral neck based on the position indicated by the navigation system. The acetabulum was then under-reamed by 1 mm and the trial cup was inserted using the press-fit technique. The target cup placement angle was 40° of anatomical inclination. The cup anteversion was adjusted according to the required ACPL. Finally, femoral rasping was performed. The final rasp was used for the examination. Femoral anteversion was matched to the native femoral anteversion using the navigation system. When stem anteversion was changed, it was increased or decreased by 20° with respect to the native femoral anteversion. We used a trial head of 32 mm in diameter, and the neck size was selected so that the postoperative leg-length discrepancy and global offset compared with the contralateral side were almost same in each case according to the navigation system. After THA, a seat-type pressure sensor (I-SCAN, Nitta, Osaka, Japan) for real-time monitoring was placed between the iliopsoas muscle and anterior pelvic wall or anterior cup edge using an ilioinguinal approach (Fig. 1A,B). We used one sensor seat per sample. The sensor seat for each specimen was calibrated in advance under the same conditions.

### Assessment of the surface pressure of the iliopsoas muscle

After placement of the trial implant and seat-type pressure sensor, we evaluated the surface pressure of the iliopsoas muscle. First, we measured its surface pressure when the ACPL was 0 mm, 3 mm, and 6 mm (Fig. 1C,D,E,F, respectively). The largest value of the ACPL (6 mm) in this study was set with reference to the smallest previously reported value of the mean ACPL in symptomatic IPI patients (5.8 mm)^12^, and the value of 3 mm was set to be half this. The anterior cup protrusion was visible to the naked eye (Fig. 1C). Therefore, we measured ACPL using digital caliper (measurement range: 0.01–150 mm) in each evaluation. Each evaluation was performed with the hip in 20° and 10° of extension, the neutral position, and 10° of flexion. During each measurement, the position of the hip was confirmed by the navigation system to be neutral in terms of the abduction and rotation angles. In addition, the hip was kept in a stable flexed position by placing pillows between the femur and the surgical table. This method has been reported as a means to control all three rotational degrees of freedom of the hip with high reproducibility^19^. The femoral stem, neck size, and head size were fixed during these evaluations in each specimen. Second, we evaluated the surface pressure of the iliopsoas muscle when stem anteversion was varied as follows: native femoral anteversion and increased and decreased by 20° compared with the native femoral anteversion. While varying the anteversion, the ACPL was kept at 0 mm. Finally, we evaluated the surface pressure of the iliopsoas muscle when the stem offset was varied by changing the neck-shaft angle of the stem. Increased offset was defined as a 125° neck-shaft angle of the femoral stem. Decreased offset was defined as a 135° neck-shaft angle of the stem. In the decreased offset condition, the neck size was − 3 mm compared with that in the increased offset condition to adjust the leg length discrepancy. The maximum and mean surface pressure of the iliopsoas muscle were measured twice in each setting and average score was recorded and analyzed.

### Statistical analysis

The Shapiro–Wilk test showed that our data were not normally distributed. ACPL and stem anteversion data were analyzed using the Friedman test in each setting. Based on the results of the Friedman tests, pairwise tests were performed using the Wilcoxon signed-rank test. Stem offset data were analyzed using the Wilcoxon signed rank test. All statistical analyses were performed using SPSS for Windows version 27 (IBM Corp., Armonk, NY, USA). A *p* value of less than 0.05 was considered statistically significant.

Given the small number subjects, a post hoc calculation of effect size and statistical power was performed with G*power software (version 3.1.9.7) using the data for the maximum surface pressure with the hip in 20° and 10° of extension when the ACPL was changed. Based on the calculated effect size, the statistical power was 0.822 in 20° of extension and 0.844 in 10° of extension, indicating that 10 specimens would be sufficient for detecting statistically significant differences.

## Results

### Anterior cup protrusion length

**Figure 2:**
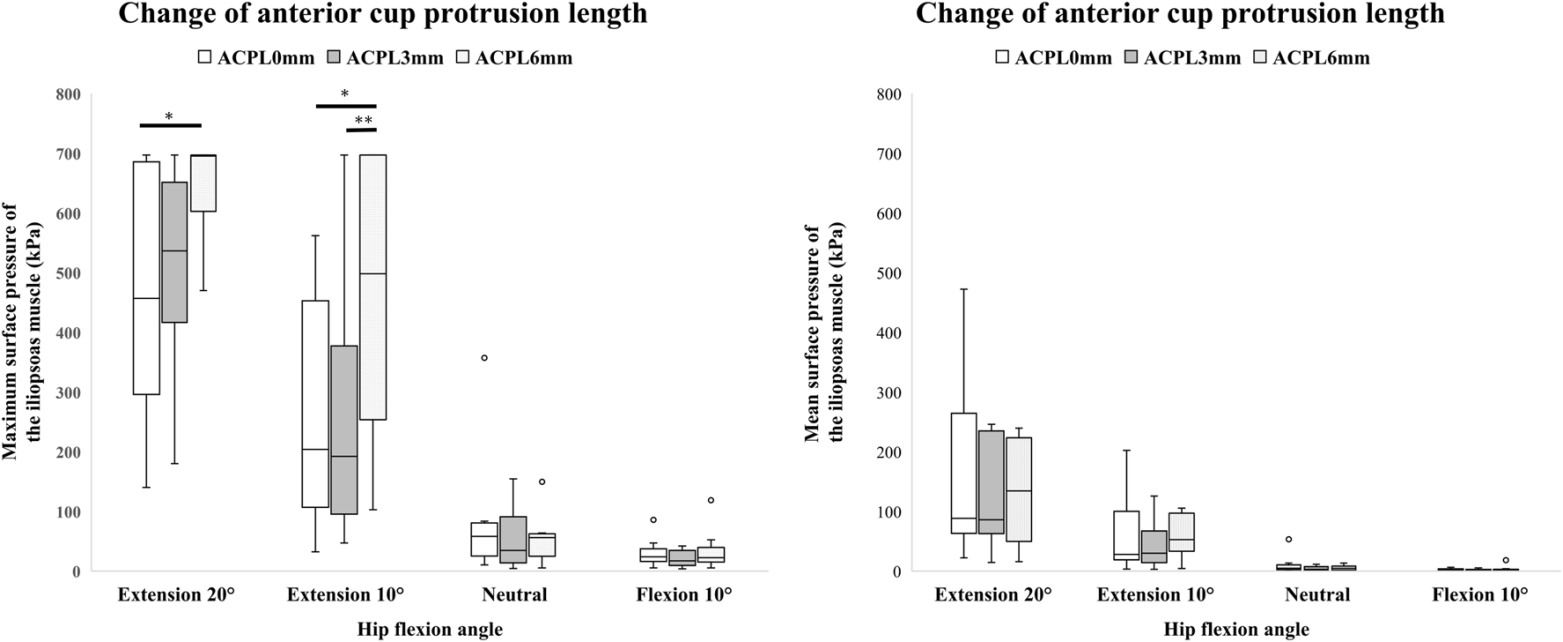
Maximum and mean surface pressure of the iliopsoas muscle when the anterior cup protrusion length (ACPL) was changed. *p < 0.05, **p < 0.01.

The changes in the maximum and mean surface pressure of the iliopsoas muscle when the ACPL was 0 mm, 3 mm, and 6 mm are summarized in Fig. 2, Tables 1 and 2. The maximum surface pressure with the hip in 20° of extension was highest when the ACPL was 6 mm, showing a significant difference between 0 and 6 mm (*p* = 0.014), but not between 0 and 3 mm (*p* = 0.576) or between 3 and 6 mm (*p* = 0.057). The maximum surface pressure with the hip in 10° of extension was significantly higher when the ACPL was 6 mm compared with 0 mm and 3 mm (*p* = 0.010 and 0.002, respectively). With the hip in the neutral position and 10° of flexion, the maximum surface pressure was extremely low, with no significant differences according to ACPL. Mean surface pressure showed no statistically significant differences according to ACPL or hip flexion angle.

**Table 1:** Maximum surface pressure (kPa) of the iliopsoas muscle when ACPL was changed.

ACPL, mm	20° of extension			10° of extension			Neutral			10° of flexion		
	Median	Range	*P* value	Median	Range	*P* value	Median	Range	P-value	Median	Range	*P* value
0	456.6	139.8–696.9		203.8	32.1–561.8		57.9	10.3–357.0	0.209	23.9	5.2–85.6	0.146
3	536.3	179.9–696.9	0.576	191.9	47.0–696.9	0.576	34.6	4.3–154.5		17.05	3.4–41.9	
6	695	469.8–696.9	0.014	497.8	102.8–696.9	0.010	56.1	5.2–149.6		22.5	5.2–118.6	

Values are shown as the median and range. *P* value are from the Friedman test. For *p* values of < 0.05 in the Friedman test, the individual *p* values are shown for comparison with 0 mm based on the results of the post hoc test. ACPL, anterior cup protrusion length.

**Table 2:** Mean surface pressure (kPa) of the iliopsoas muscle when ACPL was changed.

ACPL, mm	20° of extension			10° of extension			Neutral			10° of flexion		
	Median	Range	*P* value	Median	Range	*P* value	Median	Range	*P* value	Median	Range	*P* value
0	88.2	22.0–472.2	0.905	27.8	3.2–201.9	0.061	4.4	0.4–53.0	0.497	2.3	0.6–6.4	0.078
3	86.0	14.1–245.7		29.7	2.8–125.6		3.0	0.2–11.4		2.0	0.2–5.3	
6	134.1	15.7–239.2		52.5	4.1–105.3		4.6	0.3–13.4		1.5	0.1–18.3	

Values are shown as the median and range. *P* value are from the Friedman test. For *p* values of < 0.05 in the Friedman test, individual *p* values are shown for comparison with 0 mm based on the results of the post hoc test. ACPL, anterior cup protrusion length.

### Stem anteversion

**Figure 3:**
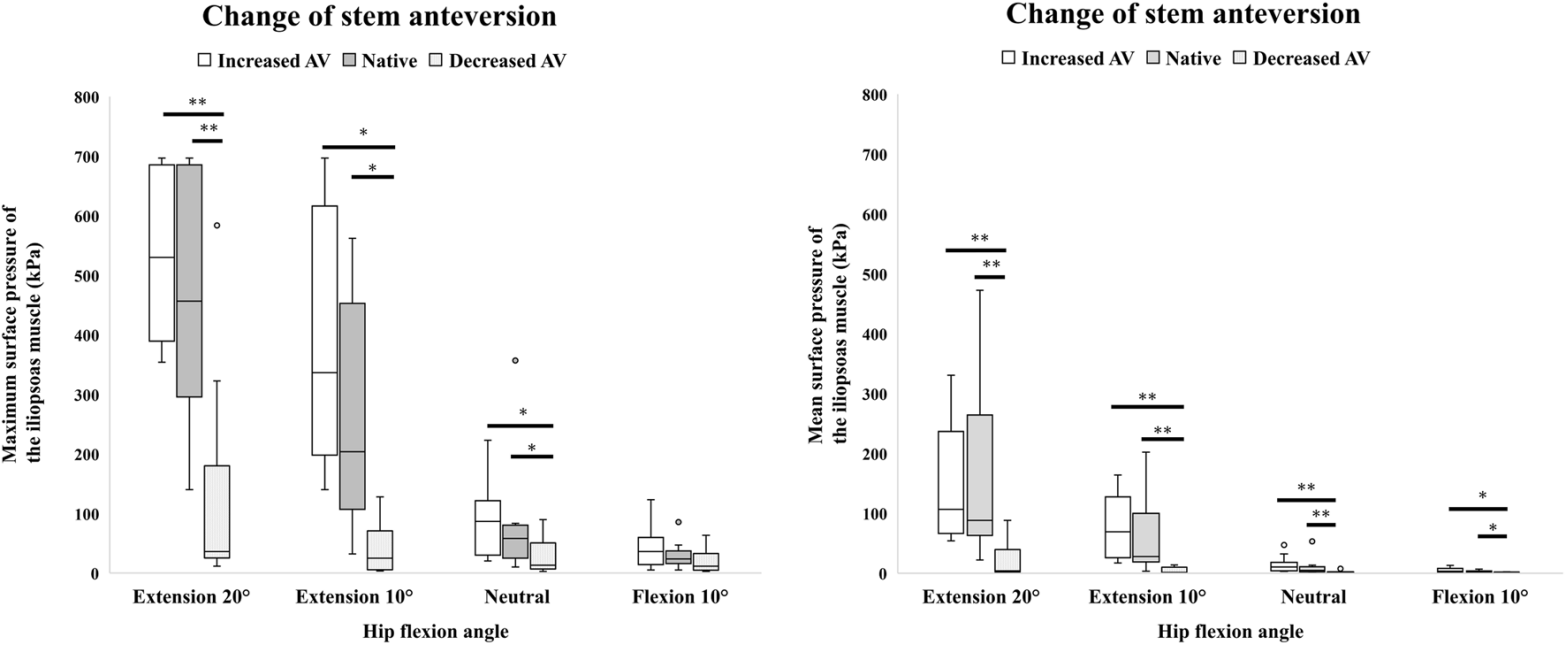
Maximum and mean surface pressure of the iliopsoas muscle when the stem anteversion (AV) was changed. *p < 0.05, **p < 0.01.

Changes in the maximum and mean surface pressure of the iliopsoas muscle when stem anteversion was varied are summarized in Fig. 3, Tables 3 and 4. Increased stem anteversion showed a tendency to increase both the maximum and mean surface pressure in all hip position except for the mean surface pressure with the hip in 20° of extension, but these increases were not significant compared with native anteversion. On the other hand, decreased stem anteversion resulted in statistically significant decreases in both the maximum and mean surface pressure compared with native anteversion for all hip positions except for the maximum surface pressure with the hip in 10° of the flexion.

**Table 3:** Maximum surface pressure (kPa) of the iliopsoas muscle when stem anteversion was changed.

	20° of extension			10° of extension			Neutral			10° of flexion		
	Median	Range	*P* value	Median	Range	*P* value	Median	Range	*P* value	Median	Range	*P* value
Increased Anteversion	529.8	353.9–696.9	0.82	336.6	139.8–696.9	0.074	86.8	20.3–222.9	0.502	36.1	5.2–123.2	0.285
Native anteversion	456.6	139.8–696.9		203.8	32.1–561.8		57.9	10.3–357.0		23.9	5.2–85.6	
Decreased anteversion	36.1	11.4–583.7	0.001	25.0	3.4–127.9	0.014	13.3	2.7–89.8	0.007	11.4	2.7–63.6	

Values are shown as the median and range. *P* value are from the Friedman test. For *p* values of < 0.05 in the Friedman test, individual *p* values are shown for comparison with native anteversion based on the results of the post hoc test.

**Table 4:** Mean surface pressure (kPa) of the iliopsoas muscle when stem anteversion was changed.

	20° of extension			10° of extension			Neutral			10° of flexion		
	Median	Range	*P* value	Median	Range	*P* value	Median	Range	*P* value	Median	Range	*P* value
Increased anteversion	106.4	54.0–250.4	0.655	68.8	16.9–163.9	0.18	10.1	2.9–47.0	0.18	3.4	0.3–13.0	0.823
Native anteversion	88.2	22.0–472.2		27.8	3.2–201.9		4.4	0.4–53.0		2.3	0.6–6.4	
Decreased anteversion	3.8	1.5–88.0	0.002	1.2	0.2–13.6	0.007	0.6	0.1–7.4	0.007	1.0	0.1–2.3	0.025

Values are shown as the median and range. *P* value are from the Friedman test. For *p* values of < 0.05 in the Friedman test, individual *p* values are shown for comparison with native anteversion based on the results of the post hoc test.

### Stem offset

**Figure 4:**
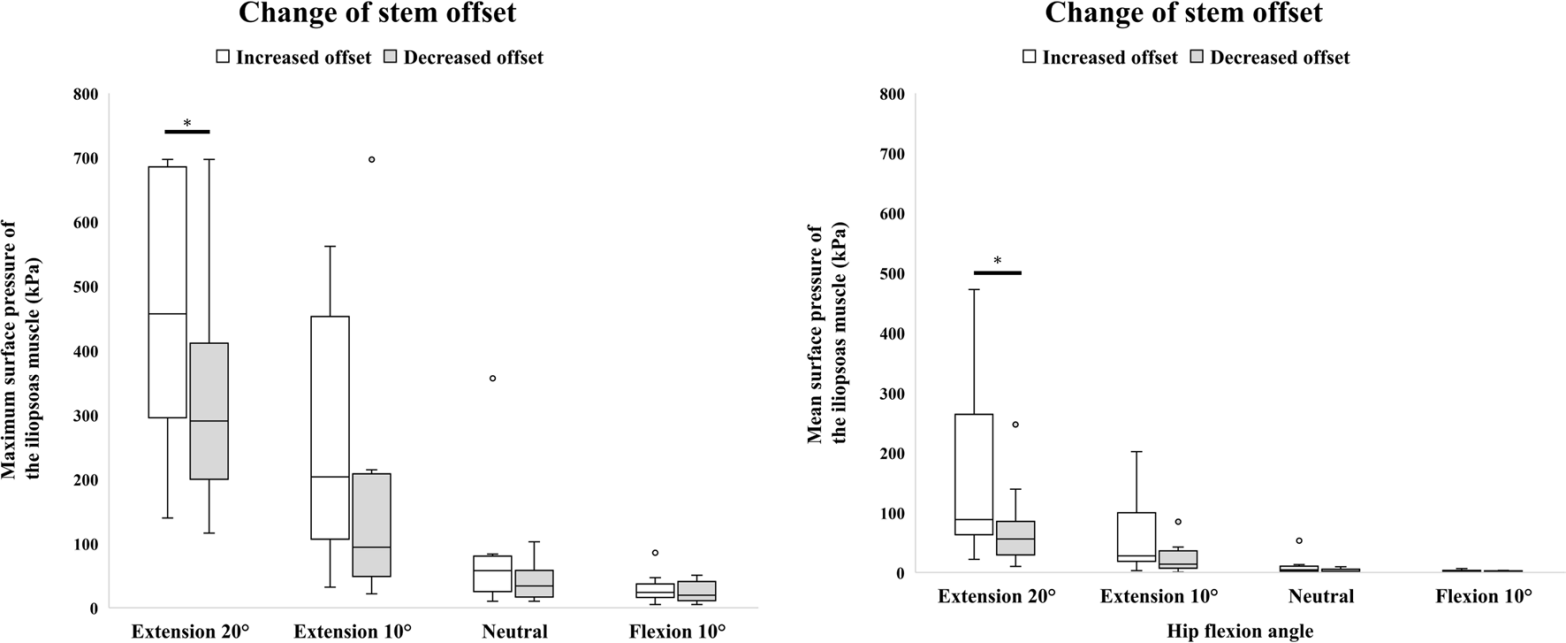
Maximum and mean surface pressure of the iliopsoas muscle when the stem offset was changed. *p < 0.05, **p < 0.01.

Changes in the maximum and mean surface pressure of the iliopsoas muscle when the stem offset was varied are summarized in Fig. 4, Tables 5 and 6. The maximum and mean surface pressure of the iliopsoas muscle was significantly increased with increased stem offset compared with decreased stem offset in 20° of extension (*p* = 0.012 and *p* = 0.021, respectively). In other hip positions, there were no significant differences.

**Table 5:** Maximum surface pressure (kPa) of the iliopsoas muscle when stem offset was changed.

	20° of extension			10° of extension			Neutral			10° of flexion		
	Median	Range	*P* value	Median	Range	*P* value	Median	Range	*P* value	Median	Range	*P* value
Increased offset	456.6	139.8–696.9	0.012	203.8	32.1–561.8	0.066	57.9	10.3–357.0	0.093	23.9	5.2–85.6	0.594
Decreased offset	29.1	116.3–696.9		94.2	21.7–696.9		34.0	10.3–102.8		19.7	5.2–50.6	

Values are shown as the median and range. The Wilcoxon signed-rank test was used for comparisons between stem offset values.

**Table 6:** Mean surface pressure (kPa) of the iliopsoas muscle when stem offset was changed.

	20° of extension			10° of extension			Neutral			10° of flexion		
	Median	Range	*P* value	Median	Range	*P* value	Median	Range	*P* value	Median	Range	*P* value
Increased offset	88.2	22.0–472.2	0.021	27.8	3.2–201.9	0.051	4.4	0.4–53.0	0.050	2.3	0.6–2.0	0.139
Decreased offset	56.0	10.3–247.2		13.8	0.7–84.9		2.1	0.4–9.6		1.3	0.5–3.4	

Values are shown as the median and range. The Wilcoxon signed-rank test was used for comparisons between stem offset values.

## Discussion

In this study, we directly measured the maximum and mean surface pressure of the iliopsoas muscle after THA to determine the relationship between the surface pressure of the iliopsoas muscle and implant alignment. A small ACPL (3 mm) had a smaller effect on the maximum surface pressure of the iliopsoas muscle, but a large ACPL (6 mm) significantly increased the maximum surface pressure of the iliopsoas muscles compared with ACPL of 0 mm during hip extension. In addition, decreased stem anteversion resulted in significant decreases in the maximum and mean surface pressure of the iliopsoas muscle compared with native femoral anteversion. Furthermore, a large stem offset resulted in significant increases in the maximum and mean surface pressure of the iliopsoas muscle compared with decreased stem offset in 20° of extension.

The reported ACPL for symptomatic IPI ranges from 2 to 27 mm in the axial plane on CT scans^7,10,12^. However, the mean ACPL has been reported to be 0 mm to 17 mm in the axial plane even in patients who are asymptomatic after THA^7,10,15,16^. Thus, the overlap of ACPL between symptomatic and asymptomatic IPI is wide, ranging from 2 to 17 mm. Despite general agreement that large ACPL causes symptomatic IPI, the association between small ACPL and symptomatic IPI remains unclear^7,10,12,15,16^. We consider the minimum ACPL value associated with symptoms to be the most clinically relevant. Therefore, the present study focused on smaller ACPL values within the range of overlap between symptomatic and asymptomatic patients. We compared ACPL of 0 mm and 6 mm with the hip in 20° and 10° of extension, finding that the maximum surface pressure significantly increased when ACPL was 6 mm even though the mean surface pressure of the iliopsoas muscle tended to remain the same or decrease. When the ACPL was 6 mm, higher pressure was recorded at the protruding part of the cup, while relatively low pressure was recorded elsewhere, resulting in a lower average pressure (Fig. 1F). Similar changes occurred for comparison between ACPL of 0 mm and 3 mm, but the amount of change was relatively small. These findings suggest that symptomatic IPI is caused by strong localized stimulation of the iliopsoas muscle at the protruding part of the cup, rather than pressure applied over the entire iliopsoas muscle. Based on the results of this study, the potentially unsafe range of ACPL was considered to be 3 mm to 6 mm, suggesting that ACPL greater than 6 mm should be avoided in the clinical setting.

The impact of stem anteversion on the pathogenesis of IPI is controversial. Qin et al. reported that stem anteversion was significantly greater in patients with IPI than in those without IPI (19.1° vs. 15.2°, *p* < 0.01)^11^. On the other hand, Ueno et al. found no significant differences in stem anteversion between patients with and without IPI (mean 25.9° vs. 28.4°, *p* = 0.38)^7^. In both of those studies, the differences in stem anteversion between the groups were relatively small. In the present study, we examined changes in surface pressure of the iliopsoas muscle by varying the stem anteversion up to 20°. Increased stem anteversion tended to increase the surface pressure of the iliopsoas muscle, but not significantly. Decreased stem anteversion significantly decreased its surface pressure. A change in stem anteversion moves the femur antero-posteriorly and rotates it. This may alter the position of the lesser trochanter, which is the attachment of the iliopsoas muscle, thereby changing the tension of the muscle. Although the differences were not always significant, our results suggest that stem version is a factor that affects the surface pressure of the iliopsoas muscle, especially decreased stem anteversion.

Increased stem offset may also be a risk factor for symptomatic IPI. Capogna et al. reported that excessive offset should be avoided as it has been implicated in the pathophysiology of IPI^17^. Bell et al. found that stem offset was greater in patients who underwent tenotomy for symptomatic IPI than in a control group^20^. In our study, the maximum and mean surface pressure of the iliopsoas muscle tended to increase with increasing stem offset in all hip position, and the differences were statistically significant in 20° of extension. This result is consistent with previous reports, indicating that increased stem offset affects the pathophysiology of iliopsoas impingement.

In the clinical setting, proper preoperative planning and surgery that is as reproducible as possible are important for surgeons to avoid large anterior cup protrusion and excessive stem offset. However, surgeons sometimes tolerate anterior cup protrusion in order to maintain adequate cup alignment in some patients, such as those with developmental dysplasia of the hip. The present study found that both cup and stem alignment were related to excessive loading of the iliopsoas muscle after THA. These results suggest the possibility that such excessive loading of the iliopsoas muscle can be mitigated by adjusting both the stem alignment and the cup alignment. In patients with developmental dysplasia of the hip and others expected to have anterior cup protrusion after THA, decreased stem anteversion or offset might be an option for reducing the excessive load on the iliopsoas muscle.

This study has several limitations. First, the sample size was small. However, power analysis indicated that 10 specimens would be sufficient for detecting statistically significant differences. Second, muscle strength cannot be examined in a cadaveric study. We evaluated the impact of implant alignment on the surface pressure of the iliopsoas muscle but could not replicate the actual clinical situation in terms of the effect on activities of daily living, muscle strength, and hip range of motion. Third, a cadaveric study cannot evaluate clinical symptoms such as pain. We objectively evaluated the excessive loading that occurs in the iliopsoas muscle in relation to implant alignment by measuring the surface pressure of the iliopsoas muscle. This could be valuable in basic research for evaluating how changes in implant alignment can cause excessive loading of the iliopsoas muscle. However, it is unclear the extent to which an increase in the surface pressure of the iliopsoas muscle corresponds to clinical symptoms. Further study is needed to evaluate the relationship between the surface pressure of the iliopsoas muscle and clinical symptoms. Fourth, the cadaveric hip specimens used in this study were normal and did not have thickened and shortened capsular ligaments, bone deformity, or joint contracture, which are typically observed in patients with osteoarthritis of the hip. Thus, our study does not fully reflect real patients who undergo THA. However, we believe that the change of the surface pressure of the iliopsoas muscle can be evaluated as an objective measure of the effect of implant alignment. Finally, in this study, measurements of ACPL, stem anteversion, and stem offset were performed systematically in that order. The order of the measurements may have affected the results, although the impact was expected to be small.

## Conclusions

In this study, we evaluated changes in the surface pressure of the iliopsoas muscle using a seat-type pressure sensor when the ACPL, stem offset, and stem anteversion were varied during THA. Not only large ACPL, but also changes in stem version and offset affected the maximum surface pressure of the iliopsoas muscle. When considering the pathophysiology of iliopsoas impingement, surgeons should pay attention to stem anteversion and offset as well as ACPL.

## Data Availability

The data generated and/or analyzed during the current study are available from the corresponding author on reasonable request.
